# A machine learning-based nomogram for predicting graft survival in allograft kidney transplant recipients: a 20-year follow-up study

**DOI:** 10.3389/fmed.2025.1556374

**Published:** 2025-04-01

**Authors:** Jiamin He, Pinlin Liu, Lingyan Cao, Feng Su, Yifei Li, Tao Liu, Wenxing Fan

**Affiliations:** ^1^Department of Nephrology, The First Affiliated Hospital of Kunming Medical University, Kunming, China; ^2^Organ Transplantation Center, The First Affiliated Hospital of Kunming Medical University, Kunming, China

**Keywords:** kidney graft survival, risk factors, LASSO regression, random survival forest, clinical prediction model

## Abstract

**Background:**

Kidney transplantation is the optimal form of renal replacement therapy, but the long-term survival rate of kidney graft has not improved significantly. Currently, no well-validated model exists for predicting long-term kidney graft survival over an extended observation period.

**Methods:**

Recipients undergoing allograft kidney transplantation at the Organ Transplantation Center of the First Affiliated Hospital of Kunming Medical University from 1 August 2003 to 31 July 2023 were selected as study subjects. A nomogram model was constructed based on least absolute selection and shrinkage operator (LASSO) regression, random survival forest, and Cox regression analysis. Model performance was assessed by the C-index, area under the curve of the time-dependent receiver operating characteristic curve, and calibration curve. Decision curve analysis (DCA) was utilized to estimate the net clinical benefit.

**Results:**

The machine learning-based nomogram included cardiovascular disease in recipients, delayed graft function in recipients, serum phosphorus in recipients, age of donors, serum creatinine in donors, and donation after cardiac death for kidney donation. It demonstrated excellent discrimination with a consistency index of 0.827. The calibration curves demonstrated that the model calibrated well. The DCA indicated a good clinical applicability of the model.

**Conclusion:**

This study constructed a nomogram for predicting the 20-year survival rate of kidney graft after allograft kidney transplantation using six factors, which may help clinicians assess kidney transplant recipients individually and intervene.

## 1 Introduction

Chronic kidney disease (CKD) is a public health problem that has a serious impact on human health (1). It is estimated that approximately 850 million people worldwide are affected by kidney diseases, a condition that now ranks as the third fastest-growing cause of death globally and is associated with substantial individual, healthcare, and societal costs (2). With the progression of CKD, patients suffering from kidney failure can only choose kidney replacement therapy to sustain their lifespan, including hemodialysis, peritoneal dialysis, and kidney transplantation (3). Kidney transplantation provides the best chance for kidney failure patients to achieve long-term, dialysis-free survival at minimal cost (4). In terms of both efficacy and financial burden, kidney transplantation is the best form of kidney replacement therapy (5). However, kidney transplant recipients are facing a risk of losing their grafts after surgery (6). Roughly one-fifth of kidney transplant recipients will suffer graft loss within 5 years, and more than half of kidney transplant recipients will suffer graft loss within 10 years (7).

The predominant etiologies of kidney graft loss are graft failure and recipient death with a functioning graft. The former may be related to surgical manipulation, immune rejection, and recurrent disease, while the most prominent causes of the latter include cardiovascular diseases (CVD), malignancies, and infections (8, 9). Kidney graft loss leads to a decline in physical function, a reduced quality of life, a greater psychological burden, and an increased risk of hospitalization and mortality (10). Additionally, it necessitates the resumption of kidney replacement therapy, exposing the patient to a higher risk of comorbidities and life-threatening complications (11). Therefore, early prediction of risk of kidney graft loss is essential for the clinical management of kidney transplant recipients, as it facilitates the improvement of both short- and long-term outcomes in these patients.

Rao et al. proposed a continuous kidney donor risk index to quantify the risk of graft failure, but their study was exclusively focused on deceased donor kidneys (12). Foucher et al. developed a composite score to predict the risk of graft loss after transplantation, but their study was limited to an 8-year follow-up period and lacked long-term survival data (13). Hernández et al. constructed a novel risk score to predict all-cause mortality after renal transplantation with no consideration of the situation of isolated graft failure with surviving recipients (14). It is challenging to accurately predict graft outcomes using traditional statistical models due to the variety of factors that influence graft survival. Habehh et al. demonstrated that machine learning techniques have made significant contributions in predicting and identifying acute health events, disease populations, disease states, and immune responses, which can address many factors and their interactions to improve the accuracy of predictive models (15). This study conducted a 20-year survival analysis of kidney graft with the aim of exploring risk factors affecting long-term survival of kidney graft and constructing a reliable and intuitive predictive model.

## 2 Materials and methods

This study follows the recommendations of the Transparent Reporting of a multivariable prognostic model for Individual Prognosis or Diagnosis (TRIPOD) (16).

### 2.1 Study population

Recipients who underwent allograft kidney transplantation at the Organ Transplantation Center of the First Affiliated Hospital of Kunming Medical University from 1 August 2003 to 31 July 2023 were included in this study. The enrollment criteria included: (1) age ≥ 18 years; (2) first kidney transplantation and single organ transplantation; (3) all donor-recipient pairs demonstrated good ABO blood group compatibility; (4) all cases underwent strict human leukocyte antigen (HLA) matching and achieved relatively favorable matching grades; (5) complement-dependent cytotoxicity (CDC) test pre-transplantation <10%; (6) recipient’s panel reactive antibody (PRA) < 10%; (7) recipients survived post-operatively with a functioning graft for at least 3 months; (8) recipients received immunosuppressive induction with biological agents (interleukin-2 receptor antagonists or lymphocyte-depleting antibodies) during the pre-, intra-, or post-transplant period, except for cases involving monozygotic twins; and (9) recipients regularly maintained on a triple immunosuppressive regimen of anti-rejection therapy after surgery: calcineurin inhibitor (CNI) + mycophenolic acid analogs + glucocorticoids. Exclusion criteria included: (1) death during surgery for transplantation; (2) insufficiency or loss of graft kidney function due to surgical factors; (3) combination of severe hematologic diseases, malignant tumors, or severe liver diseases; and (4) data missing >20%.

### 2.2 Data collection

The general characteristics of the recipients included age, gender, body mass index (BMI), primary disease (defined according to the ICD-9 diagnostic classification system, including chronic glomerulonephritis, hypertensive nephropathy, IgA nephropathy, lupus nephritis, purpuric nephritis, nephrotic syndrome, kidney stones, gouty nephropathy, polycystic kidneys, diabetic nephropathy, and unknown cause), pre-transplant dialysis modality (hemodialysis, peritoneal dialysis, mixed dialysis, and no dialysis), duration of pre-transplant dialysis, clinical comorbidities (hypertension, diabetes, CVD, and gout), smoking history, alcohol abuse history, and blood transfusion history. The laboratory data of the recipients included serum creatinine (Scr), blood urea nitrogen (BUN), serum uric acid (SUA), hemoglobin (HGB), albumin (ALB), total cholesterol (TC), triglyceride (TG), high-density lipoprotein cholesterol (HDL-C), low-density lipoprotein cholesterol (LDL-C), serum kalium (K), serum phosphorus (P), serum calcium (Ca), and blood type (A, B, O, and AB). The characteristics of the donors included age, gender, and laboratory data (Scr, BUN, SUA, and blood type). The above laboratory information was collected from the results of venous blood tests of the donor and the recipient within 24 h before surgery. Transplant-related information includes donor source, number of HLA mismatches, CDC test, PRA of recipient, whether delayed graft function (DGF) occurred after transplantation, and type of postoperative CNI (cyclosporin or tacrolimus). Donor sources include living donation between relatives (LDR), donation after brain death (DBD), and donation after cardiac death (DCD). DGF is defined as the requirement for dialysis treatment in kidney transplant recipients within 1 week postoperatively due to unrecovered kidney function (17).

### 2.3 Follow up

Kidney transplant recipients were observed in follow-up from the date of kidney transplantation, and all study subjects were observed for a minimum of 3 months, with an observation cut-off date of 31 October 2023 and an observation endpoint of kidney graft loss.

### 2.4 Definition

Kidney graft loss was defined as recipient death with a functioning graft, graft nephrectomy, resumption of dialysis again, and re-transplantation of the kidney.

### 2.5 Statistical analysis

R software (version 4.2.2; R Foundation for Statistical Computing, Vienna, Austria) and SPSS (version 26.0; IBM, Armonk, NY, United States) were employed for data analysis and graphing in this study. The difference in the analysis was statistically significant if *p* < 0.05.

Firstly, the baseline characteristics of the kidney transplant recipients and donors were statistically described and compared between groups. Missing data were addressed using multiple imputation methods. The *T*-test, Mann–Whitney U, Chi-square, and Fisher exact tests were utilized for between-group comparisons as appropriate. The overall survival rate of kidney grafts was calculated using Kaplan–Meier survival analysis.

Next, least absolute selection and shrinkage operator (LASSO) regression with 10-fold cross-validation and random survival forest (RSF) were performed to screen factors associated with the long-term survival rate of kidney graft, respectively. LASSO regression was conducted by the R package “glmnet.” RSF was performed using the R package “randomForestSRC.”

Then, the candidate variables screened by the above two machine learning methods were crossed, and the final overlapping variables obtained were subjected to Cox multivariate regression analysis to construct the prediction model. A nomogram was generated using the R packages “survival” and “rms.”

Finally, following the recommendations of the TRIPOD guidelines, the Bootstrap method (resampling = 1,000) was chosen for internal validation of the model (16). The discriminative power was assessed by the consistency index (C-index) and the area under the curve (AUC) of the time-dependent receiver operating characteristic (ROC) curve. The model calibration was evaluated by the calibration curve. The decision curve analysis (DCA) was utilized to estimate the clinical applicability.

## 3 Results

### 3.1 Patient characteristics

**Figure 1:**
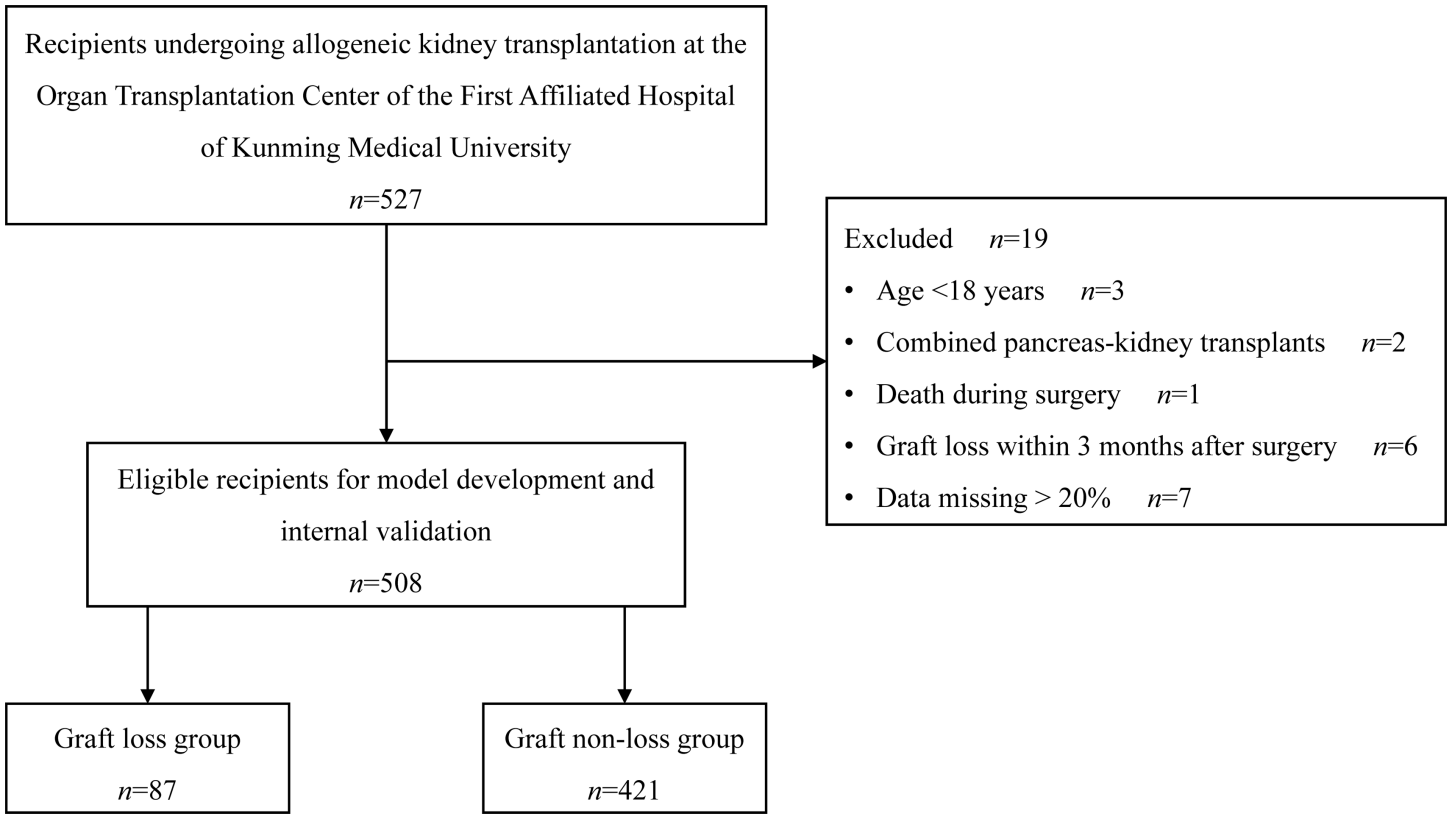
Flowchart of study enrollment.

In the beginning, 527 recipients were recruited, among whom 19 were not qualified, resulting in a final eligible population of 508 recipients. During the follow-up period, 87 recipients experienced graft loss, and 421 did not experience graft loss. Figure 1 depicts the enrollment of the study population.

The baseline characteristics are summed up in Table 1. The 508 recipients had a median age of 32.5 years (27.8, 40.0 years), consisting of 71.5% males (*n* = 363) and 28.5% females (*n* = 145). Chronic glomerulonephritis was the most common cause of kidney failure, accounting for 51.8%. The most frequent pre-transplant dialysis modality was hemodialysis, accounting for 88.2%, and the median duration of pre-transplant dialysis was 1.5 years (1.0, 2.4 years). The most common donor source was LDR (62.8%), followed by DBD (29.7%) and DCD (7.5%). The median number of HLA mismatches was 3 (2, 4). DGF occurred in 35 cases (6.9%). Cyclosporin was taken by 50 recipients (9.8%) and tacrolimus by 458 recipients (90.2%) after kidney transplantation.

**Table 1:** Characteristics between graft loss group and graft non-loss group.

Variables	Total (*n* = 508)	Graft loss group (*n* = 87)	Graft non-loss group (*n* = 421)	*p* value
Recipients				
Age (years)	32.5 (27.8, 40.0)	33.1 (25.9, 40.7)	32.5 (27.8, 39.9)	0.776
Gender, *n* (%)				0.041
Male	363 (71.5%)	70 (80.5%)	293 (69.6%)	
Female	145 (28.5%)	17 (19.5%)	128 (30.4%)	
BMI (kg/m^2^)	21.2 (19.0, 23.5)	21.4 (19.2, 23.7)	21.1 (19.0, 23.5)	0.251
Primary disease, *n* (%)				0.252
Chronic glomerulonephritis	263 (51.8%)	50 (57.5%)	213 (50.6%)	
Hypertensive nephropathy	74 (14.6%)	14 (16.1%)	60 (14.3%)	
IgA nephropathy	38 (7.5%)	5 (5.7%)	33 (7.8%)	
Lupus nephritis	8 (1.6%)	0 (0%)	8 (1.9%)	
Purpuric nephritis	22 (4.3%)	6 (6.9%)	16 (3.8%)	
Nephrotic syndrome	16 (3.1%)	1 (1.1%)	15 (3.6%)	
Kidney stones	14 (2.8%)	2 (2.3%)	12 (2.9%)	
Gouty nephropathy	21 (4.1%)	4 (4.6%)	17 (4.0%)	
Polycystic kidneys	2 (0.4%)	1 (1.1%)	1 (0.2%)	
Diabetic nephropathy	7 (1.4%)	1 (1.1%)	6 (1.4%)	
Unknown cause	43 (8.5%)	3 (3.4%)	40 (9.5%)	
Dialysis modality, *n* (%)				0.148
Hemodialysis	448 (88.2%)	79 (90.8%)	369 (87.6%)	
Peritoneal dialysis	40 (7.9%)	7 (8.0%)	33 (7.8%)	
Mixed dialysis	14 (2.8%)	0 (0.0%)	14 (3.3%)	
No dialysis	6 (1.2%)	1 (1.1%)	5 (1.2%)	
Dialysis duration (years)	1.5 (1.0, 2.4)	1.0 (0.6, 2.0)	1.5 (1.0, 3.0)	0.001
Hypertension, *n* (%)				0.265
Yes	481 (94.7%)	85 (97.7%)	396 (94.1%)	
No	27 (5.3%)	2 (2.3%)	25 (5.9%)	
Diabetes, *n* (%)				1.000
Yes	7 (1.4%)	1 (1.1%)	6 (1.4%)	
No	501 (98.6%)	86 (98.9%)	415 (98.6%)	
CVD, *n* (%)				<0.001
Yes	96 (18.9%)	37 (42.5%)	59 (14.0%)	
No	412 (81.1%)	50 (57.5%)	362 (86.0%)	
Gout, *n* (%)				1.000
Yes	26 (5.1%)	4 (4.6%)	22 (5.2%)	
No	482 (94.9%)	83 (95.4%)	399 (94.8%)	
Smoking history, *n* (%)				0.893
Yes	149 (29.3%)	25 (28.7%)	124 (29.5%)	
No	359 (70.7%)	62 (71.3%)	297 (58.5%)	
Alcohol abuse history, *n* (%)				0.905
Yes	25 (4.9%)	5 (5.7%)	20 (4.8%)	
No	483 (95.1%)	82 (94.3%)	401 (95.2%)	
Blood transfusion history, *n* (%)				0.058
Yes	413 (81.3%)	77 (88.5%)	336 (79.8%)	
No	95 (18.7%)	10 (11.5%)	85 (20.2%)	
Scr (μmol/L)	1093.3 (896.0, 1316.5)	1098.7 (906.3, 1385.0)	1093.1 (895.2, 1304.0)	0.328
BUN (mmol/L)	21.3 ± 7.2	21.5 ± 6.3	21.2 ± 7.4	0.697
SUA (μmol/L)	436.4 (363.3, 498.8)	451.0 (376.0, 516.4)	436.0 (362.5, 490.0)	0.297
HGB (g/L)	114.0 (97.0, 130.0)	101.0 (88.0, 121.0)	116.0 (99.0, 131.5)	<0.001
ALB (g/L)	43.9 (40.2, 46.6)	42.0 (36.8, 44.7)	44.3 (41.0, 46.9)	<0.001
TC (mmol/L)	3.8 (3.2, 4.3)	3.6 (3.3, 4.3)	3.80 (3.2, 4.3)	0.476
TG (mmol/L)	1.3 (1.0, 1.9)	1.3 (1.0, 2.2)	1.3 (1.0, 1.9)	0.61
HDL-C (mmol/L)	1.1 (0.9, 1.3)	1.1 (0.8, 1.3)	1.1 (0.9, 1.3)	0.323
LDL-C (mmol/L)	2.3 (1.9, 2.8)	2.2 (2.0, 2.9)	2.3 (1.9, 2.8)	0.581
K (mmol/L)	5.0 (4.4, 5.6)	5.2 (4.5, 6.0)	5.0 (4.4, 5.5)	0.032
P (mmol/L)	1.8 (1.2, 2.4)	2.2 (1.8, 2.8)	1.7 (1.2, 2.3)	<0.001
Ca (mmol/L)	2.4 (2.2, 2.5)	2.4 (2.2, 2.5)	2.4 (2.3, 2.5)	0.169
Blood type, *n* (%)				0.395
A	170 (33.5%)	25 (28.7%)	145 (34.4%)	
B	129 (25.4%)	19 (21.8%)	110 (26.1%)	
O	151 (29.7%)	31 (35.6%)	120 (28.5%)	
AB	58 (11.4%)	12 (13.8%)	46 (10.9%)	
Donors				
Age (years)	49.0 (39.0, 54.0)	49.0 (45.0, 54.0)	48.0 (38.0, 54.0)	0.033
Gender, *n* (%)				0.168
Male	223 (43.9%)	44 (50.6%)	179 (42.5%)	
Female	285 (56.1%)	43 (49.4%)	242 (57.5%)	
Scr (μmol/L)	71.2 (61.0, 83.1)	81.0 (65.0, 91.2)	69.8 (60.0, 81.0)	<0.001
BUN (mmol/L)	5.8 (4.6, 7.4)	7.7 (5.1, 9.3)	5.6 (4.5, 7.1)	<0.001
SUA (μmol/L)	300.0 (252.4, 353.0)	321.3 (233.0, 374.5)	300.0 (254.9, 346.3)	0.191
Blood type, *n* (%)				0.417
A	154 (30.3%)	22 (25.3%)	132 (31.4%)	
B	121 (23.8%)	18 (20.7%)	103 (24.5%)	
O	207 (40.7%)	42 (48.3%)	165 (39.2%)	
AB	26 (5.1%)	5 (5.7%)	21 (5.0%)	
Transplant-related				
Donor source, *n* (%)				<0.001
LDR	319 (62.8%)	43 (49.4%)	276 (65.6%)	
DBD	151 (29.7%)	19 (21.8%)	132 (31.4%)	
DCD	38 (7.5%)	25 (28.7%)	13 (3.1%)	
HLA mismatches	3 (2, 4)	3 (2, 5)	3 (2, 3)	0.023
Same blood type, *n* (%)				0.441
Yes	423 (83.3%)	70 (80.5%)	353 (83.8%)	
No	85 (16.7%)	17 (19.5%)	68 (16.2%)	
DGF, *n* (%)				<0.001
Yes	35 (6.9%)	25 (28.7%)	10 (2.4%)	
No	473 (93.1%)	62 (71.3%)	411 (97.6%)	
CNI, *n* (%)				0.079
Cyclosporin	50 (9.8%)	13 (14.9%)	37 (8.8%)	
Tacrolimus	458 (90.2%)	74 (85.1%)	384 (91.2%)	

Compared to the graft non-loss group, in terms of characteristics of recipients, the graft loss group had a greater proportion of males (*p* = 0.041), a shorter pre-transplant dialysis duration (*p* = 0.001), a higher proportion of recipients with CVD (*p* < 0.001), lower levels of HGB (*p* < 0.001) and ALB (*p* < 0.001), and higher levels of K (*p* = 0.032) and P (*p* < 0.001); regarding characteristics of donor, the age (*p* = 0.033), Scr (*p* < 0.001), and BUN (*p* < 0.001) in the graft loss group were higher; for transplant-related characteristics, the number of HLA mismatches (*p* = 0.023) and the percentage of recipients who developed DGF (*p* < 0.001) was higher in the graft loss group, and there was a statistically significant difference in donor source between the two groups (*p* < 0.001). No other baseline data differed significantly.

**Figure 2:**
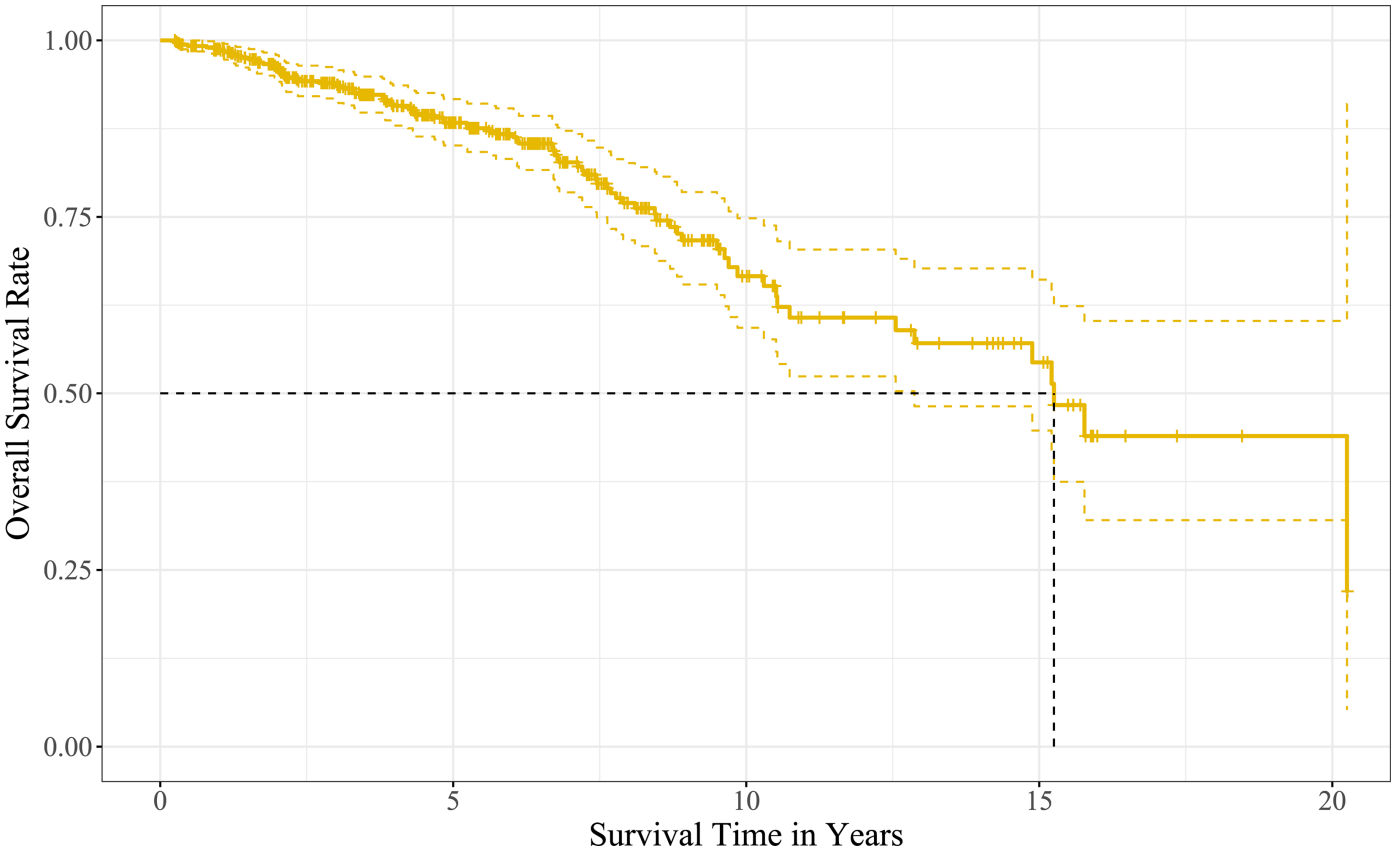
Overall survival curve of the kidney grafts after allograft kidney transplantation.

The Kaplan–Meier survival analysis showed that the median survival time of the kidney grafts in 508 recipients was 15.2 years, and the cumulative survival rates at years 1, 3, 5, 10, 15, and 20 were 98.8, 93.7, 88.3, 66.6, 54.4, and 43.9%, respectively, as described in Figure 2.

### 3.2 Characteristics selection

**Figure 3:**
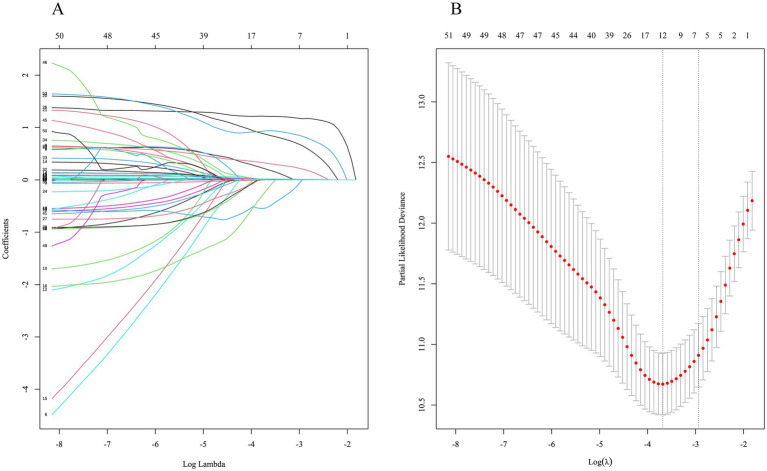
Characteristic selection by LASSO regression. **(A)** Dynamic process of variable screening. **(B)** 10-fold cross-validation to select the best *λ*.

The LASSO regression with 10-fold cross-validation screened 12 variables associated with long-term survival rates of the kidney grafts when *λ* took the minimum value (0.02512473), as displayed in Figures 3A,B. Recipient variables included gender (male), age, primary disease (polycystic kidney), CVD, P, and pre-transplant dialysis modality (no dialysis). Donor variables included age, Scr, and BUN. Transplant-related variables included donor source (DCD), number of HLA mismatches, and DGF.

**Figure 4:**
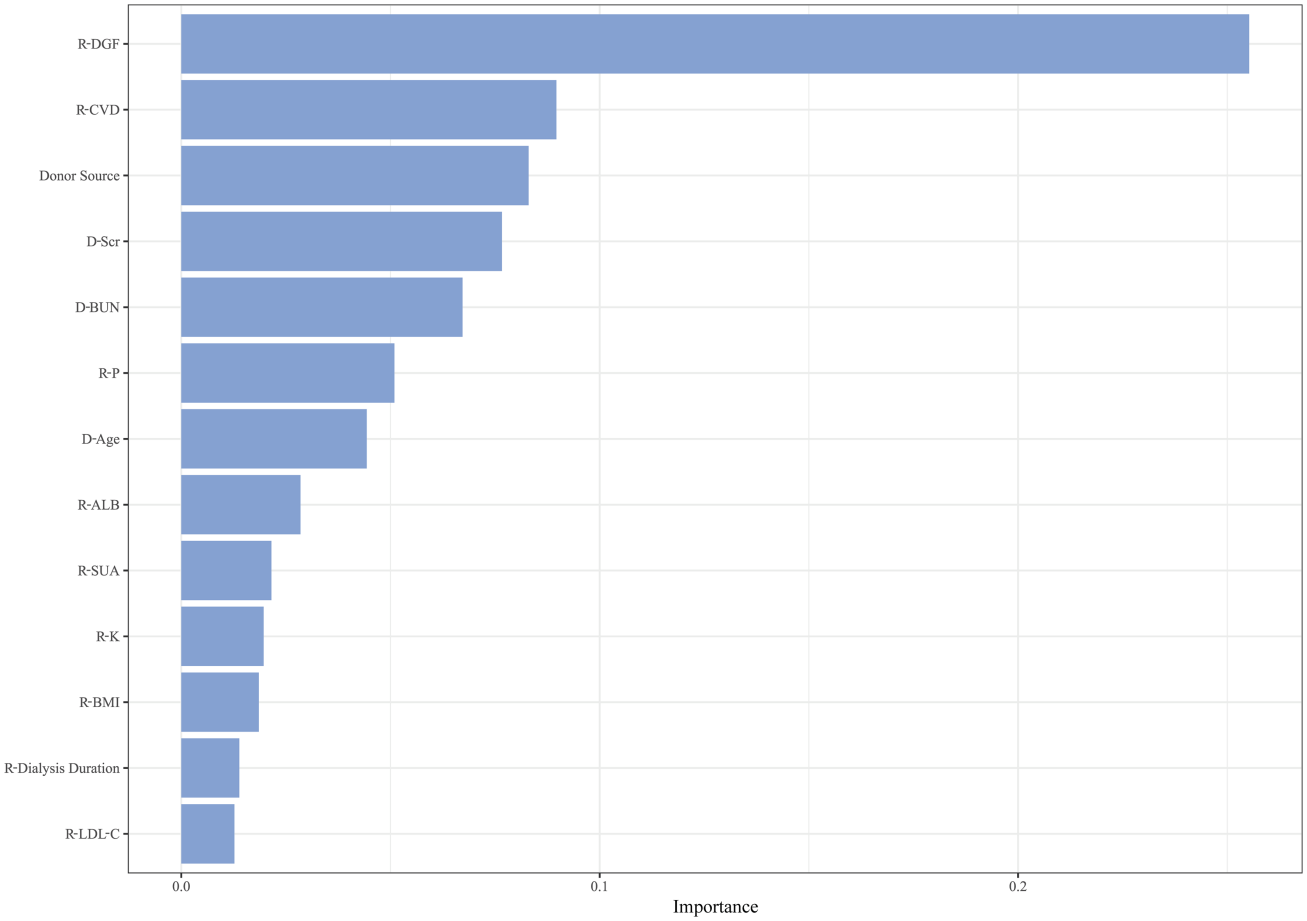
Importance ranking of variables based on RSF.

After hyperparameter tuning, RSF had the lowest prediction error rate of 19.09% when the number of trees was 80 and the number of terminal nodes was 65. There were 13 variables with significance greater than 0.01, as illustrated in Figure 4. Recipient variables included CVD, P, ALB, SUA, K, BMI, duration of pre-transplant dialysis, and LDL-C. Donor variables included Scr, BUN, and age. Transplant-related variables included donor source and DGF.

Combining the results of the two machine learning methods above, seven overlapping variables were selected as candidates for constructing the prediction model, including recipient combined CVD, DGF, recipient P, donor age, donor Scr, donor BUN, and donor source.

### 3.3 Model construction

Seven candidate variables were included in the Cox multivariate regression analysis, which revealed that recipient combined CVD (HR = 3.241, 95%CI: 1.947 ~ 5.395, *p* < 0.001), DGF (HR = 2.799, 95% CI: 1.540 ~ 5.086, *p* = 0.001), recipient P (HR = 1.605, 95%CI: 1.212 ~ 2.125, *p* = 0.001), donor age (HR = 1.037, 95%CI: 1.012 ~ 1.063, *p* = 0.004), donor Scr (HR = 1.023, 95%CI: 1.014 ~ 1.034, *p* < 0.001), and donation after DCD (HR = 3.350, 95%CI: 1.838 ~ 6.106, *p* < 0.001) were independent risk factors affecting the long-term survival rate of kidney graft, as presented in Table 2.

**Table 2:** A Cox multivariate regression analysis of kidney graft loss after allograft kidney transplantation.

Variables	HR	HR (95% *CI*)	*p* value
Recipient combined CVD	3.241	1.947, 5.395	<0.001*
DGF	2.799	1.540, 5.086	0.001*
Recipient P	1.605	1.212, 2.125	0.001*
Donor age	1.037	1.012, 1.063	0.004*
Donor Scr	1.023	1.014, 1.034	<0.001*
Donor BUN	0.997	0.924, 1.076	0.936
Donor source			
LDR		Reference	
DBD	1.237	0.640, 2.388	0.527
DCD	3.350	1.838, 6.106	<0.001*

*: *p* < 0.05.

**Figure 5:**
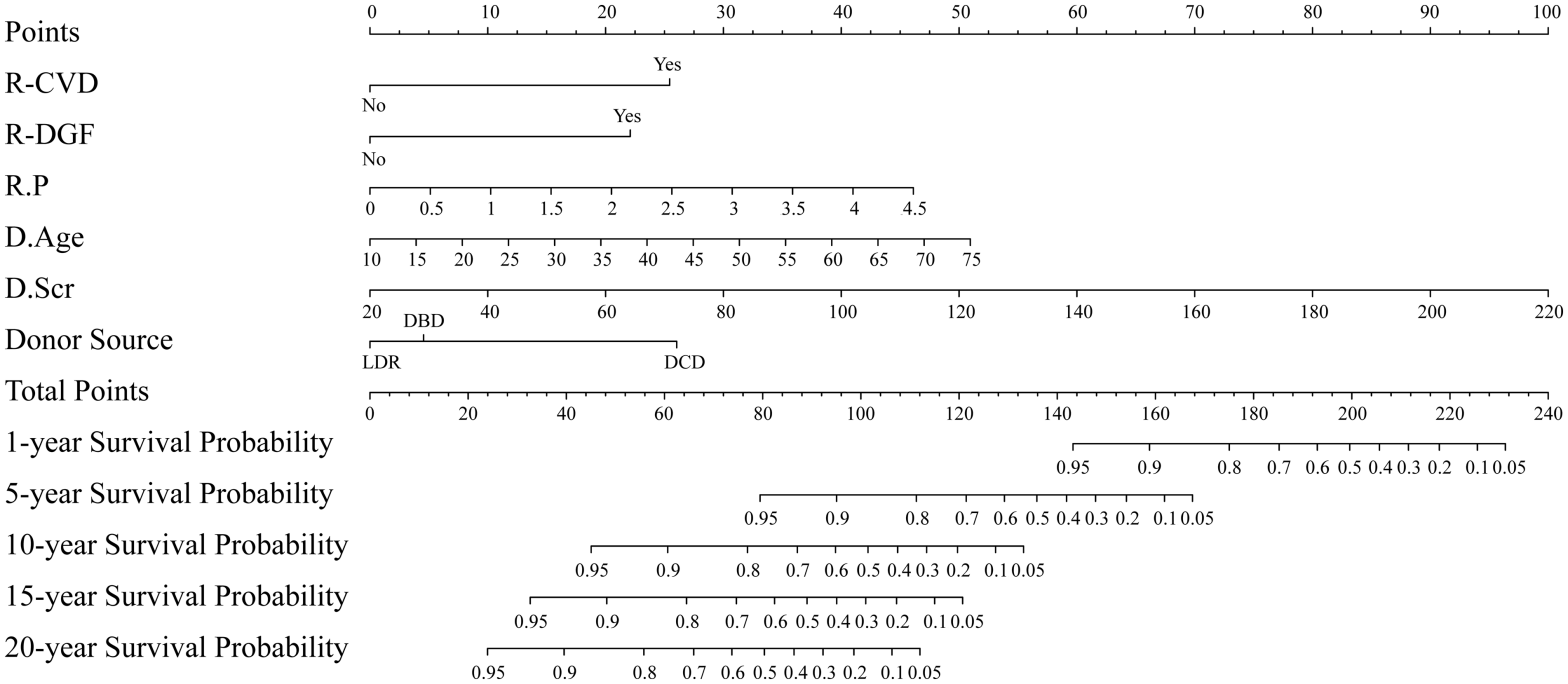
Nomogram for predicting the probability of survival of the kidney graft. R: kidney transplant recipient; D: kidney transplant donor.

A nomogram model was constructed based on the six independent risk factors mentioned above, which can predict the probability of survival of the kidney graft at 1, 5, 10, 15, and 20 years after kidney transplantation, as displayed in Figure 5.

### 3.4 Model performance and Bootstrap internal validation

**Figure 6:**
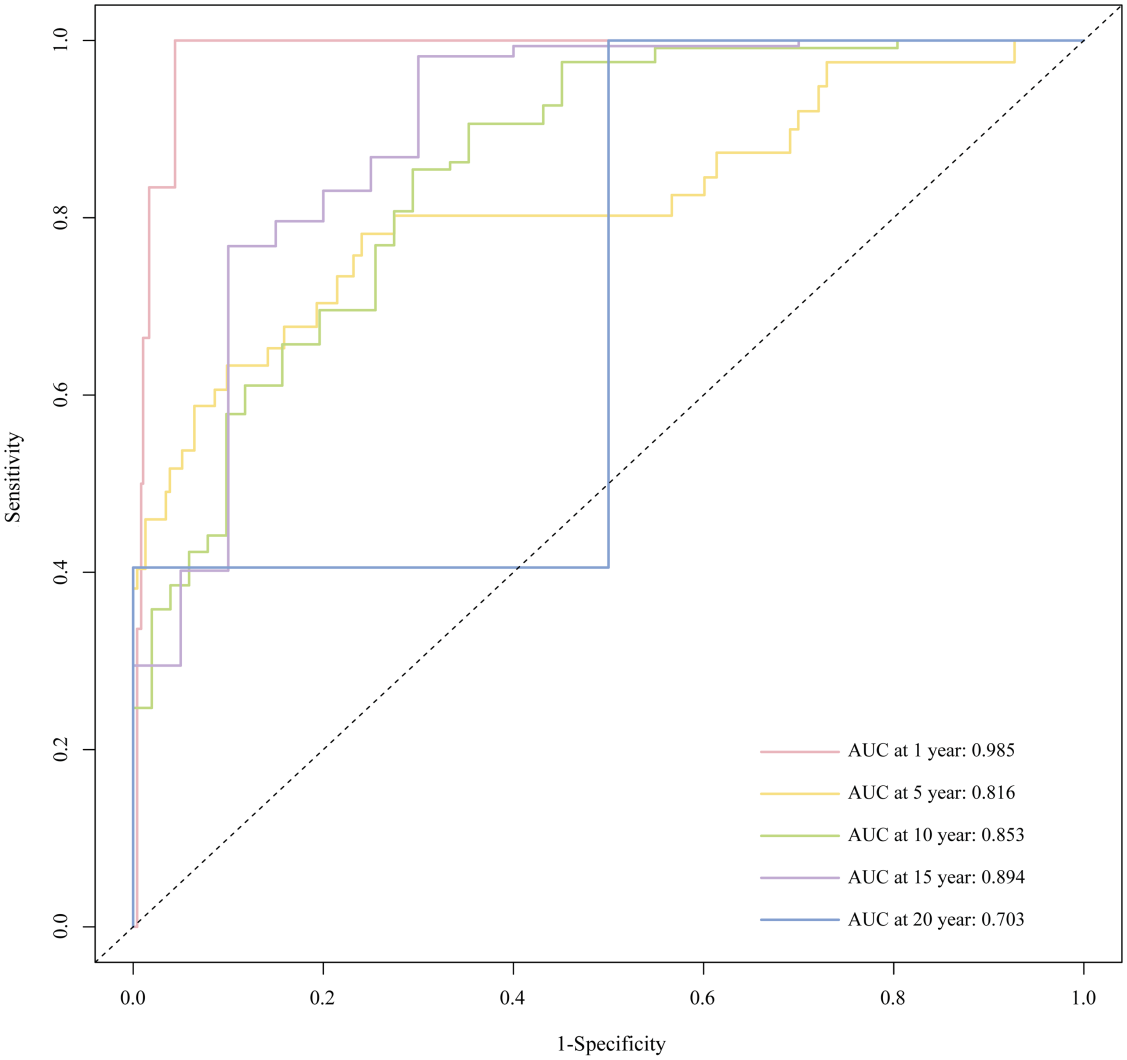
Time-dependent ROC curve of the nomogram.

With Bootstrap internal validation, the C-index was 0.827. The AUC of the ROC curve at 1, 5, 10, 15, and 20 years were 0.985, 0.816, 0.853, 0.894, and 0.703, respectively, as shown in Figure 6. It demonstrated that the model had good discriminatory ability.

**Figure 7:**
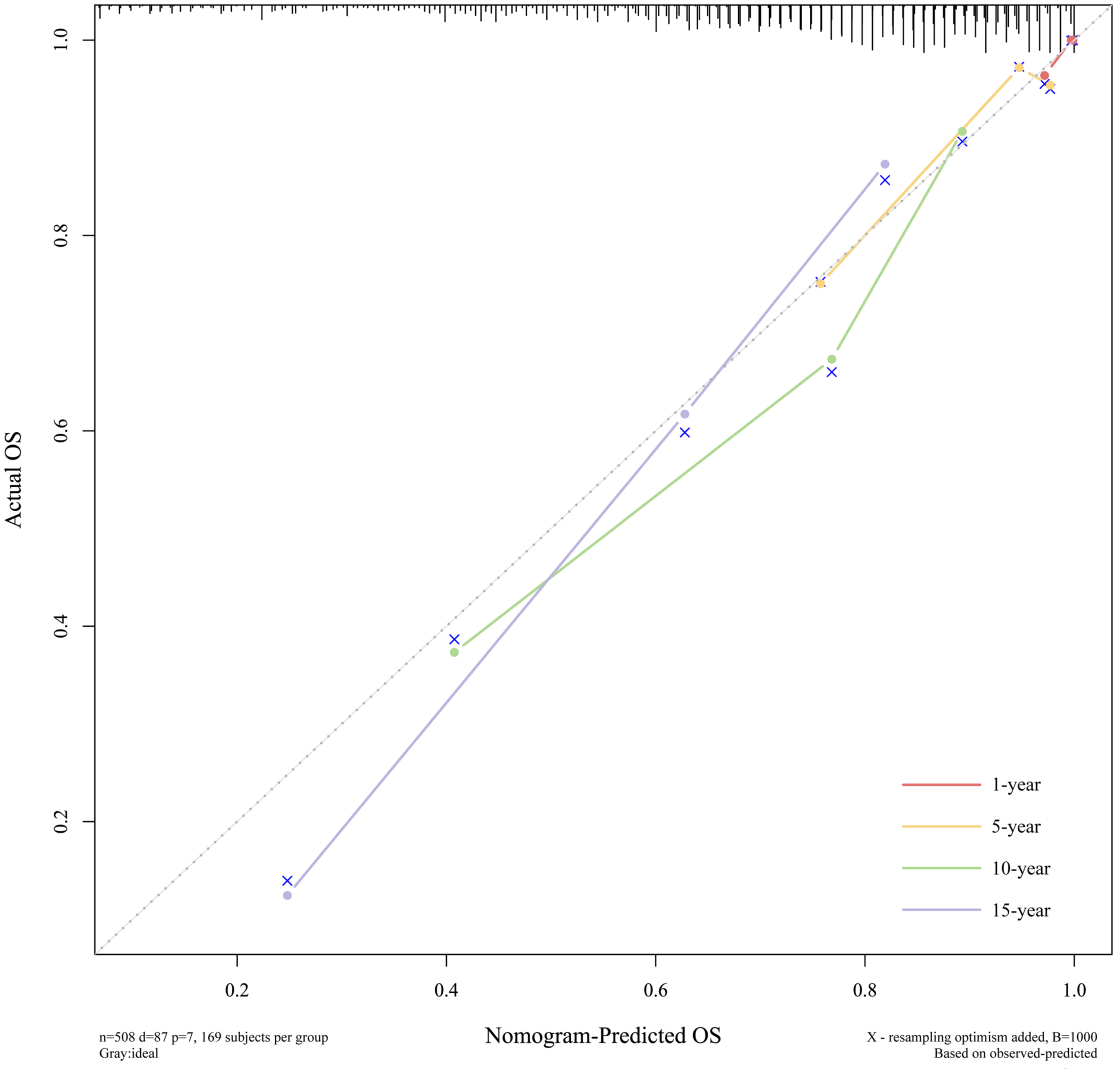
Calibration curves of the nomogram.

The calibration curves displayed excellent consistency between predictions and observations at 1, 5, 10, and 15 years, which indicated that the model had excellent calibration, as illustrated in Figure 7.

**Figure 8:**
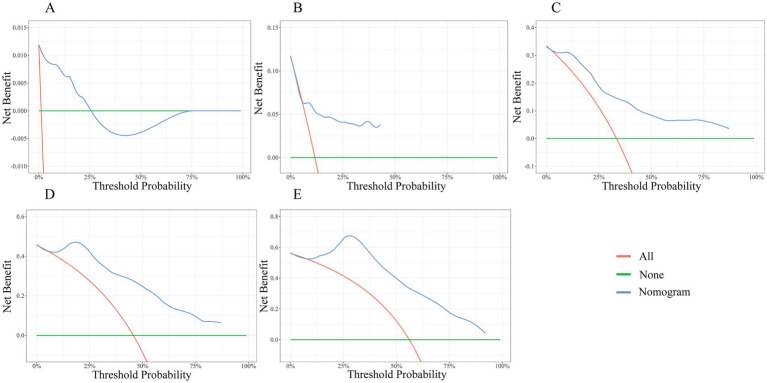
DCA of the nomogram. **(A)** 1-year; **(B)** 5-year; **(C)** 10-year; **(D)** 15-year; **(E)** 20-year.

The DCA was demonstrated in Figures 8A–E. The green horizontal line represents no endpoint event for all subjects and no intervention. Red lines represent all subjects with an endpoint event and intervention for all. The blue line represents the nomogram in this study. As shown, the blue line is essentially above the green and red lines at 5, 10, 15, and 20 years, suggesting a significant net benefit and positive clinical applicability of the model at 5, 10, 15, and 20 years. While the DCA indicates clinical utility, real-world implementation requires prospective validation of the model’s impact on actual patient outcomes and healthcare costs.

## 4 Discussion

This study conducted a 20-year follow-up of recipients who underwent allograft kidney transplantation and constructed a nomogram model for predicting the long-term survival rate of kidney graft. In this study, LASSO regression and RSF were employed for predictor screening. LASSO regression addresses multicollinearity among covariates by applying an L1-norm penalty controlled through the regularization parameter *λ*, thereby optimizing model complexity and preventing overfitting. The RSF algorithm, a machine learning approach based on an ensemble of decision trees, can deal with nonlinear relationships and interactions, analyze high-dimensional data better, and ultimately enhance the model’s predictive accuracy and stability.

As the nomogram illustrates, the kidney graft with the following features has a lower probability of survival: recipient combined CVD, recipient developing DGF, recipient with a higher P level, donor with older age, donor with a higher Scr level, and donation after DCD. Given that the factors presented above are readily available in practice, this user-friendly nomogram may be helpful for clinical decision-making. For example, suppose a kidney transplant recipient combined CVD and DGF, with a P of 2 mmol/L, whose donor is from a 30-year-old DCD, and whose donor end-stage Scr is 120 μmol/L. Using the nomogram, the corresponding points for each predictor are assigned as follows: 25 points for CVD, 22 points for DGF, 20 points for *p* = 2 mmol/L, 15 points for donor age of 30 years, 50 points for donor terminal Scr = 120 μmol/L, and 26 points for donation after DCD. The total score was 158 points by adding up the above six scores. By drawing a vertical line down the corresponding scale on the “Total Points” line segment, the predicted graft survival probabilities are 90% at 1 year, 25% at 3 years, and less than 5% at 10, 15, and 20 years post-transplantation. Clinicians can readily utilize this nomogram to estimate long-term graft survival probabilities for individual kidney transplant recipients, enabling the identification of high-risk populations and facilitating early targeted interventions.

Research has indicated that CVD is an important cause of death and graft failure in kidney transplant recipients (18). It may be attributed to the hemodynamic disorders in CVD patients and reduced renal perfusion as well as even ischemia (19). Prolonged or severe hypoperfusion of the kidneys may accelerate the reduction of the number of normal nephrons, leading to the deterioration of kidney function (20). The common risk factors for CVD, such as hypertension and dyslipidemia, are widespread in kidney transplant recipients. In addition, long-term immunosuppression can affect the body’s metabolism and increase the risk of dyslipidemia and post-transplant diabetes (21, 22). Accordingly, clinicians can slow the progression of CVD in recipients combined with CVD by individually adjusting the immunosuppressive treatment regimen, improving lifestyle, and pharmacological interventions to control blood pressure, blood glucose, and blood lipids (23).

DGF is the commonest early complication after kidney transplantation (24). A meta-analysis demonstrated that DGF may lead to graft failure and acute rejection and is associated with deterioration in short- and long-term survival of the kidney graft (25). The effect may be related to the damage caused by ischemia–reperfusion, which can lead to cellular metabolic disorders, inflammatory reactions, and fibrosis (26). In addition, ischemia–reperfusion injury may lead to strong immune rejection of the organism, and rejection has a detrimental effect on the long-term prognosis of the graft (27, 28). Clinical factors that have been identified in existing studies to be strongly associated with DGF include surgical factors, donor kidneys from dead donors, BMI of recipients, number of HLA mismatches, HLA antibodies, pre-transplantation dialysis modalities, and so on (29–31). Clinicians should assess the risk of DGF in patients before transplantation and intervene early to avoid DGF.

Disturbances in Ca and P metabolism are prevalent in kidney failure patients (32). High levels of P in kidney transplant recipients have been demonstrated to be strongly correlated with poorer graft survival rates (33). Current clinical guidelines recommend regular monitoring of serum calcium, phosphate, and parathyroid hormone levels in kidney transplant recipients during the post-transplant period (34). High levels of P impair vascular endothelial function, induce cellular stress, premature senescence, and apoptosis, and promote arterial calcification, thereby facilitating the progression of kidney disease, increasing the risk of cardiovascular events, and ultimately affecting graft survival (35–37). Moreover, hyperphosphatemia leads to compensatory elevation of fibroblast growth factor 23 and parathyroid hormone, which can cause left ventricular hypertrophy, renal anemia, and immune dysfunction (38). Therefore, clinicians should pay attention to monitoring the P levels and regulating Ca-P metabolic disorders through pharmacological interventions and dietary control.

Previous research has revealed that increased donor age is one of the essential risk factors for the long-term survival rate of kidney graft (39). As the age of the donor increases, the glomerulus progressively hardens, and the nephron decreases in size and number (40). Research has reported that nephrosclerosis accounts for only 2.7% of kidney biopsies in donors under 30 years old, 58% in donors aged 60–69 years, and 73% in donors over 70 years old (41). In recent years, there has been a gradual increase in the proportion of elderly deceased donors donating kidneys, as the growing demand for transplants far outstrips the number of kidneys available for grafting (42). A multicenter clinical study found that although increasing donor age has always been an adverse influence on graft survival, the long-term kidney graft survival rate from elderly donor kidneys has improved significantly with advances in medical care compared to a decade ago (43). The use of elderly donor kidneys is unavoidable in the current situation of kidney shortage. Therefore, it is more critical to quantify the risks associated with elderly donor kidneys and to enhance individualized post-transplant care.

Scr is the most frequently used index for assessing kidney function, and a higher-than-normal donor Scr may represent an abnormality in the kidney function of the donor. The findings of Lenain et al. demonstrated that a deceased donor-supplied kidney accompanied by acute kidney injury was associated with a decreased long-term survival rate of kidney graft (44). The results of Torreggiani et al. revealed that good preoperative kidney function in the donor was closely associated with good postoperative kidney function in the recipient (45). John et al. showed that donor kidney function can influence graft and recipient survival in living kidney transplantation (46). Consequently, it is necessary for clinicians to quantify the impact of donor Scr on the long-term prognosis of the kidney graft for individualized care.

In this study, DCD was an independent risk factor for the long-term survival rate of kidney graft compared to LDR, whereas DBD and LDR had comparable effects. It may be explained by the fact that cardiac deaths are at a higher risk of organ ischemia, which is more likely to adversely affect the long-term prognosis of kidney graft (47). It has been demonstrated that fibrosis is more likely to be detected in the kidneys of recipients undergoing DCD compared to DBD when performing a renal biopsy at 1 year postoperatively (48). Nonetheless, Won et al. suggested that DCD donor kidneys have an acceptable overall survival despite their detrimental effect on survival (49). Moreover, it was also noted in a study that there was no significant difference in short- and long-term kidney graft survival between DCD and DBD (50). This is inconsistent with the results of this study. Therefore, further prospective, multicenter, large-scale clinical studies are warranted to evaluate the long-term prognostic impact of DCD and DBD on kidney graft outcomes. What is certain, nevertheless, is that living donor kidneys have a higher survival rate of kidney graft and living donor kidney transplantation is the best treatment for kidney failure (51).

There have been some related studies on predicting the prognosis of kidney transplantation before. Hernández et al. developed a predictive model using Cox regression analysis (14). Oomen et al.’s study combined literature review, expert opinions, and multivariate logistic regression to construct a predictive model for predicting graft function (52). This study integrates LASSO regression and the Random Survival Forest (RSF) algorithm with Cox regression, which helps mitigate model overfitting, enables effective analysis of high-dimensional data, and enhances the predictive accuracy and stability of the model.

Recipient age, recipient HGB, recipient ALB, donor and recipient BMI, number of HLA mismatches, and primary disease leading to kidney failure have also been found to be predictors of long-term kidney graft survival rate after allograft kidney transplantation in previous studies (52–54). Of these, recipient HGB, recipient ALB, and HLA mismatch numbers were also found to be statistically different between groups when analyzing the baseline data in this study. But due to the limited sample size of this study, they were not included in the final prediction model in the subsequent analyses. So, it is necessary to conduct more research to validate and optimize the model.

This study has the following shortcomings: (1) This study is a retrospective observational study with data from only a single medical institution, limiting its generalizability to patients from other regions. (2) The model was only internally cross-validated using the Bootstrap method and lacked external validation. More efforts are necessary in the future to perform external validation across multi-center cohorts to optimize the model’s generalizability and clinical applicability. (3) Patients with more than 20% missing data were eliminated from the analysis, which caused some selection bias since the data were not considered to be missing randomly. However, LASSO regression was performed to assure the accuracy of the model and to avoid overfitting. (4) Due to the limited sample size of this study, HLA mismatches—widely recognized as a crucial factor influencing kidney graft outcomes—were not incorporated into the model. Therefore, more prospective, multicenter, large-sample clinical data are needed to further optimize and validate the model in the future.

## 5 Conclusion

This 20-year follow-up study constructed and internally validated a nomogram prediction model based on a machine learning approach for predicting long-term kidney graft survival rate after allograft kidney transplantation. The nomogram has six clinical and laboratory parameters, including recipient combined CVD, occurrence of DGF, recipient P, donor age, donor Scr, and donation after DCD. The model was internally validated with excellent results and may be beneficial for clinicians in clinical management for kidney transplant recipients.

## Data Availability

The raw data supporting the conclusions of this article will be made available by the authors, without undue reservation.

## References

[1] GBD Chronic Kidney Disease Collaboration. Global, regional, and national burden of chronic kidney disease, 1990–2017: a systematic analysis for the global burden of disease study 2017. Lancet. (2020) 395:709–33. doi: 10.1016/S0140-6736(20)30045-3, PMID: 32061315 PMC7049905

[2] Francis A Harhay MN Ong ACM Tummalapalli SL Ortiz A Fogo AB. Chronic kidney disease and the global public health agenda: an international consensus. Nat Rev Nephrol. (2024) 20:473–85. doi: 10.1038/s41581-024-00820-6, PMID: 38570631

[3] Chander S Luhana S Sadarat F Parkash O Rahaman Z Wang HY. Mortality and mode of dialysis: meta-analysis and systematic review. BMC Nephrol. (2024) 25:1. doi: 10.1186/s12882-023-03435-4, PMID: 38172835 PMC10763097

[4] Lentine KL Pastan S Mohan S Reese PP Leichtman A Delmonico FL. A roadmap for innovation to advance transplant access and outcomes: a position statement from the National Kidney Foundation. Am J Kidney Dis. (2021) 78:319–32. doi: 10.1053/j.ajkd.2021.05.007, PMID: 34330526

[5] Burgan CM Summerlin D Lockhart ME. Renal transplantation: Pretransplant workup, surgical techniques, and surgical anatomy. Radiol Clin North Am. (2023) 61:797–808. doi: 10.1016/j.rcl.2023.04.003, PMID: 37495288

[6] Hariharan S Israni AK Danovitch G. Long-term survival after kidney transplantation. N Engl J Med. (2021) 385:729–43. doi: 10.1056/NEJMra2014530, PMID: 34407344

[7] Saran R Robinson B Abbott KC Agodoa LYC Bragg-Gresham J Balkrishnan R. US renal data system 2018 annual data report: epidemiology of kidney disease in the United States. Am J Kidney Dis. (2019) 73:A7–8. doi: 10.1053/j.ajkd.2019.01.001, PMID: 30798791 PMC6620109

[8] Gaston RS Fieberg A Helgeson ES Eversull J Hunsicker L Kasiske BL. Late graft loss after kidney transplantation: is "death with function" really death with a functioning allograft? Transplantation. (2020) 104:1483–90. doi: 10.1097/TP.0000000000002961, PMID: 31568212

[9] Ying T Shi B Kelly PJ Pilmore H Clayton PA Chadban SJ. Death after kidney transplantation: an analysis by era and time post-transplant. J Am Soc Nephrol. (2020) 31:2887–99. doi: 10.1681/ASN.2020050566, PMID: 32908001 PMC7790214

[10] Davis S Mohan S. Managing patients with failing kidney allograft: many questions remain. Clin J Am Soc Nephrol. (2022) 17:444–51. doi: 10.2215/CJN.14620920, PMID: 33692118 PMC8975040

[11] Kabani R Quinn RR Palmer S Lewin AM Yilmaz S Tibbles LA. Risk of death following kidney allograft failure: a systematic review and meta-analysis of cohort studies. Nephrol Dial Transplant. (2014) 29:1778–86. doi: 10.1093/ndt/gfu205, PMID: 24895440

[12] Rao PS Schaubel DE Guidinger MK Andreoni KA Wolfe RA Merion RM. A comprehensive risk quantification score for deceased donor kidneys: the kidney donor risk index. Transplantation. (2009) 88:231–6. doi: 10.1097/TP.0b013e3181ac620b, PMID: 19623019

[13] Foucher Y Daguin P Akl A Kessler M Ladrière M Legendre C. A clinical scoring system highly predictive of long-term kidney graft survival. Kidney Int. (2010) 78:1288–94. doi: 10.1038/ki.2010.232, PMID: 20861817

[14] Hernández D Sánchez-Fructuoso A González-Posada JM Arias M Campistol JM Rufino M. A novel risk score for mortality in renal transplant recipients beyond the first posttransplant year. Transplantation. (2009) 88:803–9. doi: 10.1097/TP.0b013e3181b4ac2f, PMID: 19920780

[15] Habehh H Gohel S. Machine learning in healthcare. Curr Genomics. (2021) 22:291–300. doi: 10.2174/1389202922666210705124359, PMID: 35273459 PMC8822225

[16] Collins GS Reitsma JB Altman DG Moons KG. Transparent reporting of a multivariable prediction model for individual prognosis or diagnosis (TRIPOD): the TRIPOD statement. Br J Surg. (2015) 102:148–58. doi: 10.1002/bjs.9736, PMID: 25627261

[17] Bi Q Wu JY Qiu XM Li YQ Yan YY Sun ZJ. Identification of potential necroinflammation-associated necroptosis-related biomarkers for delayed graft function and renal allograft failure: a machine learning-based exploration in the framework of predictive, preventive, and personalized medicine. EPMA J. (2023) 14:307–28. doi: 10.1007/s13167-023-00320-w, PMID: 37275548 PMC10141843

[18] Wyld MLR De La Mata NL Masson P O'Lone E Kelly PJ Webster AC. Cardiac mortality in kidney transplant patients: a population-based cohort study 1988-2013 in Australia and New Zealand. Transplantation. (2021) 105:413–22. doi: 10.1097/TP.0000000000003224, PMID: 32168042

[19] Verbrugge FH Guazzi M Testani JM Borlaug BA. Altered hemodynamics and end-organ damage in heart failure: impact on the lung and kidney. Circulation. (2020) 142:998–1012. doi: 10.1161/CIRCULATIONAHA.119.045409, PMID: 32897746 PMC7482031

[20] Verbrugge FH. Utility of urine biomarkers and electrolytes for the Management of Heart Failure. Curr Heart Fail Rep. (2019) 16:240–9. doi: 10.1007/s11897-019-00444-z, PMID: 31741232

[21] Rangaswami J Mathew RO Parasuraman R Tantisattamo E Lubetzky M Rao S. Cardiovascular disease in the kidney transplant recipient: epidemiology, diagnosis and management strategies. Nephrol Dial Transplant. (2019) 34:760–73. doi: 10.1093/ndt/gfz053, PMID: 30984976

[22] Yim SH Kim HJ Ro H Ryu JH Kim MG Park JB. Benefits of statin therapy within a year after kidney transplantation. Sci Rep. (2024) 14:2002. doi: 10.1038/s41598-024-52513-6, PMID: 38263253 PMC10805738

[23] Andersson C Hansen D Sørensen SS McGrath M McCausland FR Torp-Pedersen C. Long-term cardiovascular events, graft failure, and mortality in kidney transplant recipients. Eur J Intern Med. (2024) 121:109–13. doi: 10.1016/j.ejim.2023.10.026, PMID: 37903704

[24] Swanson KJ Muth B Aziz F Garg N Mohamed M Bloom M. Kidney delayed graft function after combined kidney-solid organ transplantation: a review. Transplant Rev (Orlando). (2022) 36:100707. doi: 10.1016/j.trre.2022.100707, PMID: 35659158

[25] Li MT Ramakrishnan A Yu M Daniel E Sandra V Sanichar N. Effects of delayed graft function on transplant outcomes: a Meta-analysis. Transplant Direct. (2023) 9:e1433. doi: 10.1097/TXD.0000000000001433, PMID: 36700066 PMC9835896

[26] Barreda P Miñambres E Ballesteros MÁ Mazón J Gómez-Román J Gómez Ortega JM. Controlled donation after circulatory death using Normothermic regional perfusion does not increase graft fibrosis in the first year Posttransplant surveillance biopsy. Exp Clin Transplant. (2022) 20:1069–75. doi: 10.6002/ect.2022.0171, PMID: 36718005

[27] Salguero J Chamorro L Gómez-Gómez E de Benito P Robles JE Campos JP. Kidney survival impact of delayed graft function depends on kidney donor risk index: a single-center cohort study. J Clin Med. (2023) 12:6397. doi: 10.3390/jcm12196397, PMID: 37835040 PMC10573826

[28] Nakamura K Kageyama S Kupiec-Weglinski JW. Innate immunity in ischemia-reperfusion injury and graft rejection. Curr Opin Organ Transplant. (2019) 24:687–93. doi: 10.1097/MOT.0000000000000709, PMID: 31592839 PMC7428085

[29] Pontrelli P Simone S Rascio F Pesce F Conserva F Infante B. Pre-transplant expression of CCR-2 in kidney transplant recipients is associated with the development of delayed graft function. Front Immunol. (2022) 13:804762. doi: 10.3389/fimmu.2022.804762, PMID: 35371047 PMC8967482

[30] Zhao H Alam A Soo AP George AJT Ma D. Ischemia-reperfusion injury reduces Long term renal graft survival: mechanism and beyond. EBioMedicine. (2018) 28:31–42. doi: 10.1016/j.ebiom.2018.01.025, PMID: 29398595 PMC5835570

[31] Motter JD Jackson KR Long JJ Waldram MM Orandi BJ Montgomery RA. Delayed graft function and acute rejection following HLA-incompatible living donor kidney transplantation. Am J Transplant. (2021) 21:1612–21. doi: 10.1111/ajt.16471, PMID: 33370502 PMC8016719

[32] Kritmetapak K Losbanos L Berent TE Ashrafzadeh-Kian SL Algeciras-Schimnich A Hines JM. Hyperphosphatemia with elevated serum PTH and FGF23, reduced 1,25(OH)_2_D and normal FGF7 concentrations characterize patients with CKD. BMC Nephrol. (2021) 22:114. doi: 10.1186/s12882-021-02311-3, PMID: 33784965 PMC8011073

[33] Sawinski D Lindner H Fitzsimmons R Shults J Locke JE Cohen JB. Dialysis nonadherence and kidney transplant outcomes: a retrospective cohort study. Am J Kidney Dis. (2022) 80:46–54. doi: 10.1053/j.ajkd.2021.09.011, PMID: 34673160 PMC9016084

[34] Ketteler M Block GA Evenepoel P Fukagawa M Herzog CA McCann L. Executive summary of the 2017 KDIGO chronic kidney disease-mineral and bone disorder (CKD-MBD) guideline update: what's changed and why it matters. Kidney Int. (2017) 92:26–36. doi: 10.1016/j.kint.2017.04.006, PMID: 28646995

[35] Hu MC Moe OW. Phosphate and cellular senescence. Adv Exp Med Biol. (2022) 1362:55–72. doi: 10.1007/978-3-030-91623-7_7, PMID: 35288873 PMC10513121

[36] Zou J Yu Y Wu P Lin FJ Yao Y Xie Y. Serum phosphorus is related to left ventricular remodeling independent of renal function in hospitalized patients with chronic kidney disease. Int J Cardiol. (2016) 221:134–40. doi: 10.1016/j.ijcard.2016.06.181, PMID: 27400310

[37] Vogt I Haffner D Leifheit-Nestler M. FGF23 and phosphate-cardiovascular toxins in CKD. Toxins (Basel). (2019) 11:647. doi: 10.3390/toxins11110647, PMID: 31698866 PMC6891626

[38] Komaba H Fukagawa M. Phosphate-a poison for humans? Kidney Int. (2016) 90:753–63. doi: 10.1016/j.kint.2016.03.039, PMID: 27282935

[39] Gerbase-DeLima M de Marco R Monteiro F Tedesco-Silva H Medina-Pestana JO Mine KL. Impact of combinations of donor and recipient ages and other factors on kidney graft outcomes. Front Immunol. (2020) 11:954. doi: 10.3389/fimmu.2020.00954, PMID: 32528472 PMC7256929

[40] Kitai Y Nangaku M Yanagita M. Aging-related kidney diseases. Contrib Nephrol. (2021) 199:266–73. doi: 10.1159/000517708, PMID: 34343996

[41] O'Sullivan ED Hughes J Ferenbach DA. Renal aging: causes and consequences. J Am Soc Nephrol. (2017) 28:407–20. doi: 10.1681/ASN.2015121308, PMID: 28143966 PMC5280008

[42] Noble J Jouve T Malvezzi P Süsal C Rostaing L. Transplantation of marginal organs: immunological aspects and therapeutic perspectives in kidney transplantation. Front Immunol. (2020) 10:3142. doi: 10.3389/fimmu.2019.03142, PMID: 32082306 PMC7005052

[43] Echterdiek F Schwenger V Döhler B Latus J Kitterer D Heemann U. Kidneys from elderly deceased donors-is 70 the new 60? Front Immunol. (2019) 10:2701. doi: 10.3389/fimmu.2019.02701, PMID: 31827468 PMC6890834

[44] Lenain R Prouteau C Hamroun A Foucher Y Giral M Maanaoui M. Association between deceased donor acute kidney injury assessed using baseline serum creatinine Back-estimation and graft survival: results from the French national CRISTAL registry. Am J Kidney Dis. (2022) 79:164–74. doi: 10.1053/j.ajkd.2021.06.022, PMID: 34416353

[45] Torreggiani M Esposito C Martinelli E Jouve T Chatrenet A Rostaing L. Outcomes in living donor kidney transplantation: the role of Donor's kidney function. Kidney Blood Press Res. (2021) 46:84–94. doi: 10.1159/000512177, PMID: 33592619

[46] John EE Mehta S Sohal PM Sandhu JS. Predictors of short-term outcomes in living donor renal allograft recipients: a prospective study from a tertiary Care Center in North India. Cureus. (2022) 14:e28335. doi: 10.7759/cureus.28335, PMID: 36168334 PMC9501959

[47] Smith M Dominguez-Gil B Greer DM Manara AR Souter MJ. Organ donation after circulatory death: current status and future potential. Intensive Care Med. (2019) 45:310–21. doi: 10.1007/s00134-019-05533-0, PMID: 30725134

[48] van der Windt DJ Mehta R Jorgensen DR Hariharan S Randhawa PS Sood P. Donation after circulatory death is associated with increased fibrosis on 1-year post-transplant kidney allograft surveillance biopsy. Clin Transpl. (2021) 35:e14399. doi: 10.1111/ctr.14399, PMID: 34176169

[49] Kwon JH Blanding WM Shorbaji K Scalea JR Gibney BC Baliga PK. Waitlist and transplant outcomes in organ donation after circulatory death: trends in the United States. Ann Surg. (2023) 278:609–20. doi: 10.1097/SLA.0000000000005947, PMID: 37334722

[50] Gavriilidis P Inston NG. Recipient and allograft survival following donation after circulatory death versus donation after brain death for renal transplantation: a systematic review and meta-analysis. Transplant Rev (Orlando). (2020) 34:100563. doi: 10.1016/j.trre.2020.100563, PMID: 32576429

[51] Siddique AB Apte V Fry-Revere S Jin Y Koizumi N. The impact of country reimbursement programmes on living kidney donations. BMJ Glob Health. (2020) 5:e002596. doi: 10.1136/bmjgh-2020-002596, PMID: 32792408 PMC7430320

[52] Oomen L de Jong H Bouts AHM Keijzer-Veen MG Cornelissen EAM de Wall LL. A pre-transplantation risk assessment tool for graft survival in Dutch pediatric kidney recipients. Clin Kidney J. (2023) 16:1122–31. doi: 10.1093/ckj/sfad057, PMID: 37398686 PMC10310505

[53] Molnar MZ Nguyen DV Chen Y Ravel V Streja E Krishnan M. Predictive score for Posttransplantation outcomes. Transplantation. (2017) 101:1353–64. doi: 10.1097/TP.0000000000001326, PMID: 27391198 PMC5219861

[54] Kaboré R Haller MC Harambat J Heinze G Leffondré K. Risk prediction models for graft failure in kidney transplantation: a systematic review. Nephrol Dial Transplant. (2017) 32:ii68–76. doi: 10.1093/ndt/gfw405, PMID: 28206633

